# Antimicrobial Susceptibility Profiles of *Klebsiella pneumoniae* Strains Collected from Clinical Samples in a Hospital in Southern Italy

**DOI:** 10.1155/2024/5548434

**Published:** 2024-04-25

**Authors:** Biagio Santella, Mariarosaria Boccella, Veronica Folliero, Domenico Iervolino, Pasquale Pagliano, Luigi Fortino, Bianca Serio, Emilia Anna Vozzella, Luigi Schiavo, Massimiliano Galdiero, Mario Capunzo, Giovanni Boccia, Gianluigi Franci

**Affiliations:** ^1^Department of Medicine, Surgery and Dentistry “Scuola Medica Salernitana”, University of Salerno, Baronissi 84081, Italy; ^2^Department of Laboratory and Infectious Disease Sciences, Agostino Gemelli University Hospital IRCCS, Rome 00168, Italy; ^3^Department of Public Health and Infectious Diseases, Sapienza University of Rome, Rome 00185, Italy; ^4^A.O.U. San Giovanni di Dio e Ruggi D'Aragona, Salerno 84131, Italy; ^5^U.O.C. of Virology and Microbiology, University Hospital “Luigi Vanvitelli”, Naples 80138, Italy; ^6^DAI Department of Health Hygiene and Evaluative Medicine, U.O.C. Clinical Pathology and Microbiological, A.O.U. San Giovanni di Dio e Ruggi D'Aragona, Salerno 84131, Italy

## Abstract

Infections caused by antibiotic-resistant bacteria represent a serious threat to global public health. Recently, due to its increased resistance to carbapenems and *β*-lactams, *Klebsiella pneumoniae* has become one of the main causes of septicemia, pneumonia, and urinary tract infections. It is crucial to take immediate action and implement effective measures to prevent further spread of this issue. This study aims to report the prevalence and antibiotic resistance rates of *K. pneumoniae* strains isolated from clinical specimens from 2015 to 2020 at the University Hospital of Salerno, Italy. More than 3,800 isolates were collected from urine cultures, blood cultures, respiratory samples, and others. *K. pneumoniae* isolates showed broad resistance to penicillin and cephalosporins, and increased susceptibility to fosfomycin and gentamicin. Extended spectrum beta-lactamase (ESBL) isolates accounted for 20–22%. A high percentage of strains tested were resistant to carbapenems, with an average of 40% to meropenem and 44% to ertapenem. The production of ESBLs and resistance to carbapenems is one of the major public health problems. Constant monitoring of drug-resistant isolates is crucial for developing practical approaches in implementing antimicrobial therapy and reducing the spread of *K. pneumoniae* in nosocomial environments.

## 1. Introduction

Globally, antimicrobial resistance (AMR) is one of the most serious threats to public health. In 2019, over one million deaths were attributed to multidrug-resistant strains (MDRs) [1]. Furthermore, owing to the ease of transmission of MDRs, particularly in healthcare environment, it is expected that more than 5 million deaths will occur by 2030 [2, 3].

Currently, among *Enterobacteriaceae* family, *Klebsiella pneumoniae* (*K. pneumoniae*) and *Escherichia coli* are regarded as the primary opportunistic pathogens species, causing various infections such as pneumonia, urinary tract infections (UTIs), bloodstream infections (BSIs), and surgical site infection [4, 5]. Moreover, the use of medical devices, such as urinary catheters, venous catheters, and respiratory support equipment represent important risk factors in nosocomial infections promoted by opportunistic pathogen bacteria [6]. Infections produced by *K. pneumoniae* have grown over the past decade and constitute a particular concern to immunocompromised individuals [7]. Increased healthcare costs, prolonged hospitalization, untreated infections, and mortality rates are the consequences of this situation [8, 9]. Treatment failure is attributed to the ability of these species to produce extended-spectrum beta-lactamases (ESBLs) capable of neutralizing cephalosporins, used to treat Gram-negative infections, including pneumonia caused by *K. pneumoniae*. ESBLs can hydrolyze and inactivate *β*-lactam antibiotics, including penicillin, cephalosporins (first, second, and third generation), and aztreonam, but not carbapenems [10]. Recently, it has been noted that ESBL-producing bacteria show a high rate of resistance to other classes of antibiotics, such as fluoroquinolones, sulfonamides, and aminoglycosides [11]. *K. pneumoniae* has developed resistance to carbapenems through the production of enzymes (carbapenemases) that represent the first-line therapy for severe infection with ESBL-producing *K. pneumoniae*. Resistance to carbapenems is attributable to one or more carbapenemase genes (*bla*_KPC_, *bla*_NDM_, *bla*_VIM_, *bla*_OXA−48_, and *bla*_IMP−Like_), whose expression usually leads to carbapenem resistance [12]. However, since the first carbapenem-resistant strain of *K. pneumoniae* (KPC) was isolated and characterized in 1996 by Yigit et al., carbapenem-resistant strains have increased rapidly and have received considerable public interest due to the scarcity of antibiotic treatment choices [13, 14]. The limited therapy choices resulting from the inefficacy of novel beta-lactam/beta-lactamase inhibitor combos, such as ceftazidime-avibactam and meropenem/vaborbactam against NDM-producing pathogens (New Delhi metallo beta-lactamase) constituted an additional barrier [15]. *K. pneumoniae* is the most common bacterial species among the carbapenem-resistant *Enterobacteriaceae* strains, recognized by WHO as one of the most important priority pathogens (included in ESKAPE pathogens) due to reduced treatment options and potential for community spread [16]. In previous studies, the mortality associated with KPC infections was expected to range between 33% and 42% in developed countries [17]. The hospital surveillance programs aim to screen patients colonized by KPC and provide isolation of patients in the ward to limit the contagion. Recent studies reported an increase in carbapenem resistance in *K. pneumoniae* in China, from 3% in 2005 to 21% in 2017 [18]. In Europe, the KPC represents the fastest growing antibiotic resistance with a six-fold increase in the number of deaths between 2007 and 2015 [19]. Other reports from the US, England, and Argentina highlighted the rapid increase of carbapenem-resistant *Klebsiella* infections [20–22]. Surveillance programs are particularly needed for carbapenemase-producing *K*. *pneumoniae* (KPC-Kp) and *E*. *coli* (CP-Ec), which are often responsible for outbreaks, as described in the latest report of the European Antibiotic Surveillance Network (EARS-Net). Due to this increase in antibiotic resistance, several studies have been conducted around the world to monitor the antibiotic resistance of *K. pneumoniae* [23]. Furthermore, it is desirable to support genotypic tests, such as DNA microarray and PCR, for the study of resistance mechanisms, mostly present in isolates at a local level, which can improve the understanding of the molecular mechanisms underlying antibiotic resistance [24]. In Pakistan, Uddin et al. reported a high prevalence of *K. pneumoniae* MDR, of which 98% were ESBL producers; furthermore, NDM-positive isolates were resistant to newer formulations, including meropenem/vaborbactam (MEV), ceftazidime/avibactam (CZA), and ceftolozane/tazobactam (C/T) [25, 26]. In Saudi Arabia, surveillance of *K. pneumoniae* antibiotic resistance revealed an increased rate of resistance among different classes of antibiotics [27]. For example, in Aseer, several studies conducted between 2015 and 2019 showed an increased rate of resistance against penicillin and cephalosporin. The results of these investigations are useful to the clinician to choose an effective and correct treatment and to contrast the phenomenon of antibiotic resistance. The aim of this study was to evaluate the antimicrobial resistance rates of *K*. *pneumoniae* isolated from clinical specimens from 2015 to 2020 in our University Hospital to improve clinical practice and therapeutic treatment.

## 2. Materials and Methods

### 2.1. Clinical Isolate Collection

Out of 3,841 *K. pneumoniae* strains were isolated from patients admitted to the University Hospital “San Giovanni di Dio e Ruggi d'Aragona” in Salerno, from January 2015 to December 2020. We collected the demographic data, including age and gender, department unit, and the site of infections for the enrolled patients. The samples included blood, urine, bronchial-aspirate, sputum, swabs (vaginal swab, sternal swab, and wound swab), and other samples (catheters, sperm culture, ascitic fluid, abscess, and liquor). Throughout the entire study period, we made sure to exclude any cultures that contained the same pathogen isolated from a single subject in case of multiple episodes of infection. To avoid duplicity in our data, we only included nonduplicated clinical isolates that were collected during the first episode of infection in the study period. We defined duplicate isolates as isolates from the same patient with an indistinguishable pattern of susceptibilities and excluded them accordingly.

### 2.2. Species Identification and Antimicrobial Susceptibility Test

All samples were collected in sterile containers and were analyzed in the microbiology laboratory, following the European Committee on Antimicrobial Susceptibility Testing (EUCAST) guidelines. Species were grown on blood agar, chocolate agar, and MacConkey agar (bioMérieux, Marcy-l'Étoile, France) and incubated at 37°C for 18–24 hours. All the biological samples indicated were processed following the official lines for processing biological clinical samples for microbiological investigations and diagnosis of infectious diseases. Identification and antibiotic resistance profile were determined by the Vitek2 system using an identification card (ID-GN) and susceptibility cards (AST-397 and AST-379 only for urine cultures). The Vitek-2 automated system involves the preparation of the inoculum, a 0.45% saline solution in which the bacterial isolates are suspended with a standard turbidity of 0.5 McFarland used for the identification and determination of the MIC at different antibiotics (AST). The ID-GN panel contained 46 fluorometric tests that included pH change and enzymatic reaction tests subjected to a kinetic fluorescence measurement. According to the manufacturer's instructions, quality control test was performed routinely once a week, and the reference Gram-negative strains were *Klebsiella oxytoca* ATCC 700,324 and *Enterobacter* ATCC 700,323. For each antibiotic, interpretation of results was done according to EUCAST clinical breakpoints. The following antibiotics were tested: ampicillin (AMP), amoxicillin/clavulanic acid (AUG), piperacillin/tazobactam (SXT), ceftazidime (CAZ), cefotaxime (CTX), cefepime (FEP), ertapenem (ETP), meropenem (MRP), imipenem (IMP), ciprofloxacin (CIP), gentamicin (CN), tigecycline (TGC), fosfomycin (FOS), and trimethoprim/sulfamethoxazole (S/T).

### 2.3. Ethical Consideration Statement

The study was conducted according to the guidelines of the Declaration of Helsinki. Approval for this study was obtained from the authority of San Giovanni di Dio e Ruggi d'Aragona Hospital. The data retrieved were anonymous and not linked to any patient. This is a retrospective study, which involves the collection of secondary data regarding bacterial isolates and their respective antimicrobial susceptibility data. The study did not have access to any sensitive patient information, only age and gender. All analyzed data contained exam codes that replaced and anonymized sensitive patient data.

### 2.4. Statistical Analysis

The demographic data of patients, including age, gender, isolated strain(s), and drug sensitivity results, were used for the analysis. The age- and sex-standardized incidences were calculated. The chi-framework test was used to compare the differences among antibiotic sensitivities over the range of years considered in the study. A chi-square test was used to verify the possible associations between the categorical variables, while the Cochran–Armitage trend test was used to verify the existence of a trend. The existence of a trend was checked only for antibiotics that showed statistically significant differences in the distribution of resistance during the years under consideration. Considering a significance level alpha = 0.05 for both tests, therefore those associations with a *p* value <0.05 were considered statistically significant. The IBM Statistical Package for Social Sciences Version 22.00 (SPSS Inc., Chicago, IL, USA) was used for data analysis.

## 3. Results

A total of 3,841 *K*. *pneumoniae* strains were isolated from January 2015 to December 2020 at the University Hospital “San Giovanni di Dio e Ruggi d'Aragona,” Salerno, Italy. Among the patients, 50.2% were females. The analysis of incidence by age group showed that patients between 61 and 75 years were 31%, followed by the age group 40–60 and 76–95 age group (21%), while for younger patients, the incidence was lower (8–10%) (Supplementary Table 1).

Additionally, there is a higher incidence of cases in the Intensive Care Unit (19.2%), General Surgery (12.9%), Nephrology (12.2%), and Internal Medicine (9.2%). This is shown by the analysis of the distribution of diagnostic materials across hospital departments from 2015 to 2020 (refer to Supplementary Table 2).

**Figure 1:**
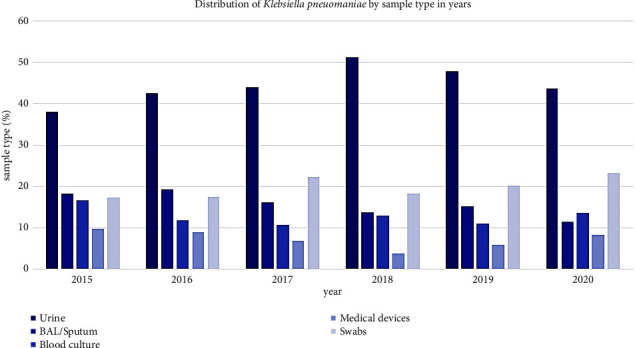
Prevalence of *Klebsiella pneumoniae* isolated from various clinical specimens. Clinical samples' detail: bronchoalveolar lavage (BAL), vaginal swab, sternal swab, wound swab (swabs), and urinary and vascular catheters (catheters).

The highest percentage of *K. pneumoniae* strains were isolated from urine samples (45.3%), followed by vaginal and wound swabs (19.9%), sputum and bronchial-aspirate samples (15.4%), blood cultures (12.5%), and medical device samples (6.9%) (Figure 1).

These data showed an increase by year of species isolated from urine samples, from 38% in 2015 to 48%–43% in 2019 and 2020, respectively.

In our study, *K. pneumoniae* showed a high rate of resistance to ampicillin (98.5%), amoxicillin/clavulanic acid (63.7%), piperacillin/tazobactam (55%), ceftazidime (61.9%), cefotaxime (64.7%), and cefepime (50.2%) (Table 1). Also, the rates of resistance to ciprofloxacin and trimethoprim/sulfamethoxazole were higher than 50%, with 62.4% and 54.3%, respectively. Moreover, a low rate of resistance was shown for the carbapenems class, with imipenem at 28%, a slightly higher rate for meropenem (39.3%) and ertapenem (42.5%).

Finally, the antibiotics that showed a resistance rate of less than 50% were tigecycline (38.8%), gentamicin (35.8%), and fosfomycin (29.2%) (Table 1).

Furthermore, some antibiotics showed a reduction of resistance rate over the years. Among them, trimethoprim/sulfamethoxazole in 2015 showed a resistance rate of 62,2% while in 2020 it was 33.5%, the same trend for tigecycline (from 45.5% in 2015 to 28.5% in 2019) and meropenem (from 49.0% in 2015 to 32.1% in 2020). On the contrary, fosfomycin showed an increase in the resistance rate from 22.7% in 2015 to 40.8% in 2020. Lastly, the ESBL isolates were between 20 and 25% each year.

## 4. Discussion

In recent decades, nosocomial infections have posed a major threat to healthcare systems worldwide, mainly in developing countries [28]. *K. pneumoniae*, being one of the most common pathogens, plays a significant role as the causative agent of severe infections [29]. As the main nosocomial opportunistic pathogen, it is continuously exposed to various antibiotics, leading to the development of resistance mechanisms and the spread of MDR strains. However, the antibiotic resistance rate of *K. pneumoniae* may vary depending on geographic location, population, and antibiotic management [30]. This study investigated the prevalence and antibiotic resistance profiles of *K. pneumoniae* isolates from different specimen sources in inpatients from 2015 to 2020. The analysis of incidence by age group revealed a low rate in pediatric patients (0–18 years), with the highest incidence in adult patients aged between 61 and 75 years (31%) and 76– and 95 years (21%). These findings align with Cassini et al., who reported the highest infection rate in Italian individuals aged 65 years or older [19]. *K. pneumoniae* was mainly isolated from urine cultures (45.3%) followed from vaginal and wound swabs (19.9%), sputum and bronchial-aspirate samples (15.4%), blood cultures (12.5%), and medical device samples (6.9%). In contrast, the study conducted by Romanus et al. showed a following distribution of 30.5% in the urinary tract, 23.6% in respiratory samples and 40% in blood cultures [31]. Another study reported a major portion of bacteria isolated from sputum and bronchoalveolar specimens (49.5%), with a higher rate than in other samples, while the isolates from urine were the second population (16.3%), followed by blood source (8.7%) [32].

In our study, we found that between 20 and 25% of ESBL *K*. *pneumoniae* isolates consistent with a previous study in Nigeria where the frequency of this phenotype was 17% [31]. It is essential to note that the prevalence of the ESBL phenotype could vary according to geographical location; for instance, this type of resistance ranges from 14% to 16% for France and England, respectively, to 5% in the United States [33]. Moreover, the Annual Epidemiological Report for 2020 in the European Union showed 33.9% of *K. pneumoniae* isolated were ESBL, and in Italy, this rate was over 50%. On the other hand, in Iran, studies by Kashefieh et al. and Kiaei et al. reported a percentage of ESBL isolates of 65% and 41.4%, respectively [34, 35]. Finally, in China, Xu et al. reported 33.7% of *K. pneumoniae* isolates were ELBS-producing [35].

The high incidence of ESBL-producing *K*. *pneumoniae* is likely due to the common consumption of third generation cephalosporins in society [36]. According to our study, the highest incidence of resistance was to ampicillin (98.5%). This result aligns with studies conducted in Iran and Russia, where the resistance rate was 97% [37, 38].

In this study, the most effective antibiotics against *K. pneumoniae* were fosfomycin, imipenem, gentamicin, and tigecycline, with susceptibility of 71%, 72%, 64%, and 61%, respectively.

Fosfomycin is a broad-spectrum antibiotic against Gram-negative and Gram-positive bacteria and has played a major role in treating MDR of *Enterobacteriaceae* in recent years [39]. Our results regarding fosfomycin for the treatment of *K. pneumoniae* align with other studies where the resistance rate was 16% and 10.9%. Multiple mechanisms of fosfomycin resistance may be attributed to antimicrobial modifying enzymes, target site modification, or reduced permeability [40].

Due to the limited availability of new antimicrobial agents, the evaluation of older antimicrobial drugs, including fosfomycin, polymyxins, and aminoglycosides, appears as a possible strategy for managing infections induced by multidrug-resistant (MDR) bacteria. Fosfomycin, discovered in 1969, has a bactericidal action against Gram-positive and Gram-negative bacterial species [41]. In recent years, fosfomycin has been increasingly used in Europe and Asia to treat infections caused by carbapenem-resistant *Enterobacteriaceae* isolates [39, 42].

However, the increased use of fosfomycin favors the spread of resistant isolates, as reported by recent studies evaluating the antimicrobial activity of fosfomycin against carbapenem-resistant *K. pneumoniae* [43, 44]. These studies report different resistance rates of KPC isolates to fosfomycin in China (61.9%), Taiwan (36.4%), Germany (32%), and the United States (15%).

However, there are few studies on the correlation between fosfomycin resistance and carbapenem resistance in *K. pneumoniae* isolates, reporting the prevalence and mechanisms of fosfomycin resistance in KPC clinical isolates.

Another effective class of antibiotics for the treatment of this opportunistic pathogen is carbapenems. With the advent of the ESBL-positive phenotype and increasing resistance to these antibiotics, carbapenems have emerged as the drugs of choice for the treatment of serious infections caused by *K. pneumoniae* ESBL [45]. In our study, the resistance rate to imipenem was 28%, consistent with other studies conducted previously in which the resistance rates were 25.7% [34, 46]. These data are concerning because it is much higher than the rate reported in Europe (10%).

Finally, another interesting class of antibiotics is represented by aminoglycosides which is currently the most widely used drugs for the treatment of multiresistant *K. pneumoniae*. In our study, the gentamicin sensitivity rate was 64%, similar to the study by Hesam et al., which showed a gentamicin resistance rate of 34.6% [47]. In contrast, in the study by Kashelfieh et al., the resistance rate was 51% [34].

## 5. Conclusions

This study, through a comprehensive statistical analysis, provided an evaluation of the antibiotic susceptibility trends of *K. pneumoniae* isolates, revealing increased sensitivity to piperacillin/tazobactam, cefepime, meropenem, imipenem and, conversely, increased resistance to fosfomycin. In conclusion, this study aims to underline the importance of monitoring the evolution of antimicrobial susceptibility models in our province, implementing surveillance systems for multiresistant microorganisms and verifying the effectiveness of the empirical therapies adopted, minimizing therapeutic failures and spread of the phenomenon of antibiotic resistance. Innovative and continuous surveillance strategies are an indispensable resource to preserve antibiotics' effectiveness and patients' health. Finally, improving hygienic conditions and prevention strategies are necessary to slow the spread of multiresistant microorganisms and infections acquired in nosocomial environments.

## Figures and Tables

**Table 1:** Resistance rates of the clinical isolates of *Klebsiella pneumoniae* to antimicrobial agents.

*Klebsiella pneumoniae*		2015	2016	2017	2018	2019	2020	^*∗*^	^*∗∗*^
Class	Antibiotic	R % (n.)	R % (n.)	R % (n.)	R % (n.)	R % (n.)	R % (n.)		
*β*-lactam/*β*-lactamase inhibitor combinations	Amoxicillin/clavulanic acid	65.7 (463)	66.1 (498)	58.7 (494)	66.6 (572)	64.6 (814)	60.3 (610)	0.026	0.206
	Piperacillin/tazobactam	64.1 (465)	63.3 (550)	53.6 (584)	47.2 (707)	56.9 (850)	48.8 (637)	<0.0001	<0.0001

Cephalosporins	Ceftazidime	61.8 (466)	65.7 (551)	60.0 (587)	64.6 (704)	64.7 (853)	54.3 (668)	0.0001	0.0319
	Cefotaxime	67.0 (466)	69.1 (551)	62.9 (587)	66.9 (707)	66.8 (840)	56.0 (680)	<0.0001	0.000452
	Cefepime	56.0 (466)	59.2 (551)	46.5 (585)	39.6 (652)	51.9 (476)	51.6 (556)	<0.0001	0.0048

Carbapenems	Ertapenem	44.3 (411)	51.2 (551)	43.9 (586)	33.1 (706)	45.4 (852)	37.4 (431)	<0.0001	0.0016
	Meropenem	49.0 (465)	48.6 (551)	39.7 (585)	30.2 (706)	40.8 (850)	32.1 (663)	<0.0001	<0.0001
	Imipenem	29.2 (465)	33.3 (540)	33.5 (585)	20.9 (651)	23.0 (478)	28.1 (570)	<0.0001	0.0021

Fluoroquinolones	Ciprofloxacin	63.7 (466)	62.3 (551)	61.2 (587)	64.8 (707)	66.4 (853)	55.5 (676)	0.0005	0.16

Aminoglycosides	Gentamicin	31.5 (466)	31.6 (551)	43.1 (587)	40.5 (707)	36.9 (852)	29.4 (660)	<0.0001	0.796

Tetracyclines	Tigecycline	45.5 (279)	52.1 (363)	37.1 (456)	30.8 (448)	28.5 (228)	NA	<0.0001	<0.0001

Others	Fosfomycin	22.7 (463)	27.9 (498)	22.9 (493)	23.2 (573)	37.0 (662)	40.8 (441)	<0.0001	<0.0001
	Trimethoprim/sulfamethoxazole	62.2 (466)	63.7 (551)	63.2 (587)	65.2 (707)	45.4 (853)	33.5 (681)	<0.0001	<0.0001

^*∗*^ *p* value with chi-square, ^*∗∗*^*p* value with Cochran–Armitage trend test.

## Data Availability

Epidemiological data used to support the results of this study are included within the article.

## Supplementary Materials

Supplementary Materials Supplementary Tables include Supplementary Table 1: distribution of cases for gender and age class and Supplementary Table 2: distribution of diagnostic materials by hospital department from 2015 to 2020.
