# Supplementary material for: Antimicrobial Susceptibility Profiles of Klebsiella pneumoniae Strains Collected from Clinical Samples in a Hospital in Southern Italy
Source: Can J Infect Dis Med Microbiol. 2024 Apr 25;2024:5548434. doi: 10.1155/2024/5548434 (PMC11065490; doi:10.1155/2024/5548434)
Supplement: Supplementary Materials — Supplementary Tables include Supplementary Table 1: distribution of cases for gender and age class and Supplementary Table 2: distribution of diagnostic materials by hospital department from 2015 to 2020. [file 5548434.f1.pdf]

**Age (Years)**	**2015**	**2016**	**2017**	**2018**	**2019**	**2020**
**0-15**	7.1	9.1	5.0	15.3	6.2	6.1
**16-30**	14.6	3.8	4.4	4.2	8.0	3.1
**31-45**	11.2	11.1	13.0	8.8	9.3	10.1
**46-60**	24.1	26.4	21.7	17.8	18.1	19.2
**61-75**	26.5	29.1	31.5	31.0	35.3	35.2
**76-95**	16.6	20.5	24.4	22.8	23.1	26.3
**Total (n.)**	465	550	585	706	851	684

**GENDER**	2015	2016	2017	2018	2019	2020
**Female**	39.4	50.4	56.8	51.7	48.5	52.3
**Male**	60.6	49.6	43.2	48.3	51.5	47.7
**Total (n.)**	465	550	585	706	851	684

Supplementary Table 1: Distribution of cases (%) for gender and Age class

Department n.(%)	2015	2016	2017	2018	2019	2020	TOT
Intensive Care Unit	119(25.6)	127(23.1)	108(18.5)	127(18.0)	137(16.1)	119(17.4)	737(19.2)
Surgery	38(8.2)	48(8.7)	79(13.5)	86(12.2)	141(16.6)	103(15.1)	495(12.9)
Nephrology and Urology	63(13.5)	54(9.8)	61(10.4)	85(12.0)	122(14.3)	84(12.3)	469(12.2)
Internal Medicine	45(9.7)	62(11.3)	58(9.9)	52(7.4)	74(8.7)	63(9.2)	354(9.2)
Cardiology	54(11.6)	61(11.1)	54(9.2)	39(5.5)	54(6.3)	58(8.5)	320(8.3)
Hematology	22(4.7)	36(6.5)	20(3.4)	36(5.1)	41(4.8)	43(6.3)	198(5.2)
Neonatology	26(5.6)	31(5.6)	19(3.2)	67(9.5)	28(3.3)	23(3.4)	194(5.1)
Anesthesia and Reanimation	2(0.4)	18(3.3)	34(5.8)	62(8.8)	44(5.2)	19(2.8)	179(4.7)
Neurology	17(3.7)	20(3.6)	26(4.4)	22(3.1)	15(1.8)	34(5.0)	134(3.5)
Infectious Diseases	19(4.1)	30(5.5)	14(2.4)	16(2.3)	19(2.2)	14(2.0)	112(2.9)
Orthopedics	19(4.1)	6(1.1)	9(1.5)	14(2.0)	7(0.8)	8(1.2)	63(1.6)
Gynecology	7(1.5)	5(0.9)	11(1.9)	6(0.8)	13(1.5)	7(1.0)	49(1.3)
Pediatrics	5(1.1)	9(1.6)	6(1.0)	7(1.0)	9(1.1)	4(0.6)	40(1.0)
Oncology	4(0.9)	2(0.4)	4(0.7)	7(1.0)	4(0.5)	9(1.3)	30(0.8)
ICU-COVID	NA	NA	NA	NA	NA	26(3.8)	26(0.7)
Otolaryngology	0	0	3(0.5)	3(0.4)	2(0.2)	3(0.4)	11(0.3)
Gynecology	0	0	0	0	1(0.1)	2(0.3)	3(0.1)
Others	25(5.4)	41(7.5)	79(13.5)	77(10.9)	140(16.5)	65(9.5)	427(11.1)
Total	465	550	585	706	851	684	3841

Supplementary Table 2: Distribution of diagnostic materials by hospital department from 2015 to 2020.ICU—Intensive Care Unit, NA—non-applicable; Others included the ophthalmology unit, gastroenterology unit, Day hospital unit and outpatient clinics.
